# Buffering PTSD in Canine Search and Rescue Teams? Associations with Resilience, Sense of Coherence, and Societal Acknowledgment

**DOI:** 10.3390/ijerph17176184

**Published:** 2020-08-26

**Authors:** Milena Kaufmann, Matthias Gelb, Mareike Augsburger

**Affiliations:** 1 Division of Psychopathology and Clinical Intervention, University of Zurich, 8050 Zurich, Switzerland; milena.kaufmann@gmail.com; 2 TCRH Training Center Rescue and Help Mosbach, 74821 Mosbach, Germany; aerztlicher-direktor@tcrh.de

**Keywords:** posttraumatic stress disorder, social acknowledgment, resilience, sense of coherence, canine search and rescue teams

## Abstract

Rescue workers present an elevated risk for posttraumatic stress disorder (PTSD) and recently, research has begun to focus on coping styles and social support as protective factors in this population. Associations in the particular group of search and rescue dog handlers still lack evidence. The aim of the study is to investigate if functional cognitions and social support also decrease the risk for PTSD. Active voluntary rescue dog handlers (*n* = 116) rated levels of resilience, sense of coherence, and social acknowledgment (SAQ; subscales general disapproval, familial disapproval, recognition), in addition to a trauma checklist and PTSD symptoms. Linear regression analyses and two different graph models were calculated to explore associations, as well as potential pathways. Controlling for trauma exposure, the SAQ general disapproval emerged as the only significant predictor in the regression model. In the graph models, SAQ familial disapproval was linked to SAQ recognition and SAQ general disapproval. The latter, together with a sense of coherence manageability, affected PTSD re-experiencing symptoms through resilience. The findings are in line with earlier work. The study underlines the importance of targeting resilience and manageability, as well as enhancing social support in prevention programs for PTSD in canine search and rescue teams. Future research is warranted to further investigate model stability and replicate findings.

## 1. Introduction

Individuals working within emergency and rescue services are frequently exposed to human suffering. This type of psychological trauma exposure was also acknowledged within the Diagnostic and Statistical Manual of Mental Disorders, Fifth Edition (DSM-5), in which job-related aversive details of a traumatic situation were explicitly described [1]. Related to frequent exposures is the high risk for the development of posttraumatic stress disorder (PTSD) in rescue workers. A recent review reported PTSD prevalence rates of up to 14.6% for rescue and emergency personnel, but rates considerably differ between professional groups and also across geographical regions [2]. In light of the inevitable exposure to job-related psychologically traumatic events, the identification of risk and resilience factors for chronic PTSD, specifically in rescue workers, is imperative for tailoring effective prevention strategies and maintaining long-term mental health.

Regarding work-related factors, a number of studies have confirmed associations with PTSD for overall traumatic load and previous traumatic experiences [3,4], long-term deployment [5], and volunteer work, in contrast to a paid employment [6]. However, not all studies consistently reported associations. In a prospective study with paramedic trainees, baseline lifetime exposure to psychologically traumatic events and exposure during the assessment period did not constitute a risk factor for PTSD at follow-up [7]. These previous findings and inconsistencies highlight individual and subjective aspects that can enhance individual vulnerability, such as peri- and posttraumatic personal cognitions, beliefs, or coping styles, as shown in a review [8]. Supporting is the finding that the subjective perception of threat to deployment-related events was the only correlate of PTSD in a sample of Portuguese firefighters when controlling for number, recency, and frequency of events [9]. Moreover, an approach-based coping style characterized by active processing of critical situations was related to higher well-being in a large and diverse sample of Swedish first responders [10]. Functional cognitions and coping styles relate to the concept of psychological resilience, which describes individuals’ cognitive and emotional resources to adapt to adverse situations and maintain mental health [11]. Numerous studies point to the importance of psychological resilience among rescue workers. Resilience was negatively associated with PTSD symptoms in a cross-sectional study with paramedics [12] and in a heterogeneous group of first responders after an earthquake in New Zealand [13]. Perceived resilience to stress at baseline was an important cognitive risk factor for PTSD symptom development two years later in a sample of paramedic trainees [7].

Sense of coherence in its revised concept might be a specific facet of resilience. The term refers to a global orientation rooted in the strong dynamic feeling of being able to integrate and balance stressors in life [14,15]. Initial research within the field of rescue work indicates that a strong sense of coherence can serve as a functional coping style in dealing with potentially traumatic situations and can also buffer the development of PTSD symptoms in ambulance personnel [12,16].

The importance of social support offered by members of both personal and work-related networks for preventing PTSD is also emphasized [8], as such social support may lead to increased resilience. A meta-analytical review confirmed its effectiveness in promoting mental health after stressful events in different rescue worker professions [17]. A beneficial effect of social support for preventing PTSD was also demonstrated in empirical studies with paramedics [3,7], as well as firefighters [18], and was shown in a recent review with mixed samples of rescue workers [19]. Studies further demonstrate that engaging with a pet leads to reduced stress and contributes to increased resilience in the face of adversity [20,21]. In addition, a study confirmed positive associations between dog affinity and perceived social support [22]. Dog owners have been shown to perceive the human–dog bond as a non-judgmental, empathic, and unconditional source of social support [21]. Anthropomorphism of their dogs is one possible explanation for this finding [23], however, the underlying mechanisms are still unclear [21].

Despite the increased scientific interest in explaining long-term mental health in rescue workers, and in light of the converging evidence regarding the impact of cognitive factors and social support on PTSD, one particular group of rescue workers lacks considerable empirical evidence, i.e., voluntary dog handlers who are working within canine search and rescue teams. There is one study investigating the mental health of US dog handlers involved in 9/11 rescue work. Whilst rates of PTSD were generally low, there was still a significant difference between 9/11 deployed and non-deployed team members. Even more crucial, low social support was associated with increased risk for PTSD [24]. Regarding European countries, research concerning voluntary canine search and rescue teams remains very scarce. One study from the Netherlands reported very low levels of PTSD in response to deployment to the Haiti earthquake in 2010 in a mixed rescue team with police dog handlers [25]. Vulnerability to deployment-related PTSD depends on the characteristics of specific occupational groups and varies significantly following exposure to the same psychologically traumatic event [26]. Other relevant factors that must be considered include geographical operation sites and characteristics of deployment [2]. For instance, lack of training and preparedness for missions, as well as structural barriers in voluntary rescue teams, are associated with poorer mental health outcomes [26,27]. In order to tailor a prevention program to reduce the risk for PTSD, it is crucial to consider specific professional groups of rescuers. In addition, recent research in Animal Assisted Intervention (AAI) highlights the beneficial effects of canine interaction on mental health among soldiers suffering from PTSD [28]. Although the setting and goals of AAI and voluntary canine search and rescue deployment are significantly different, these results suggest that the presence of canines can have a beneficial effect on PTSD. Additionally, there appears to be a strong interaction between the stress levels of dogs and their handlers [29]. Taken together, both findings highlight the particularities of voluntary dog handlers, as opposed to other rescue workers, and emphasize the necessity of taking a closer look at this particular subgroup.

To sum up, to the best of our knowledge so far, no study has investigated potential protective factors in voluntary canine search and rescue teams. The aim of the study is to investigate cognitive factors and coping styles that might decrease the risk for PTSD after exposure to psychologically traumatic events. The study provides insights into correlates of PTSD that have been previously identified as protective factors in other rescue worker samples. We hypothesized that sense of coherence, resilience, as well as social support in the form of perceived social acknowledgment were all associated with lower levels of PTSD.

## 2. Materials and Methods

The current analyses are part of a project that investigates mental health in canine search and rescue teams. Specific details about recruitment procedures and the sample are reported elsewhere [30]. The study was approved by the ethics committee of the Philosophical Faculty at Zurich University (approval No. 17.12.3).

### 2.1. Procedure

The cross-sectional study was an online survey provided to all members of the German Federal Association of Canine Search and Rescue Teams. An invitation that included a link to the survey was sent to all members with active email accounts who were subscribed to the electronic mailing list in spring 2018. At the time, the association consisted of approximately 1800 active members. Individuals could participate if they were at least 18 years old, German-speaking, and without a diagnosis of serious mental disorders (due to ethical reasons). All participants provided informed consent.

### 2.2. Participants

In total, *n* = 116 active dog handlers participated (40% men). Mean age was 43.6 years (standard deviation, SD = 11.7). The majority of the sample held a University or University of Applied Science degree (45%), had completed “Abitur” (University entrance certificate, 20%), or secondary-level education (17%), followed by completion of vocational training (14%), basic secondary school qualifications (3%), and completion of primary school (1%). Time in the canine search and rescue teams varied considerably (range: 0 to 35 years), with a mean duration of 8.9 years (*SD* = 7.1). For details, see [24].

### 2.3. Measures

#### 2.3.1. Trauma Exposure

The German version of the life event checklist (LEC-5) was applied to measure lifetime traumatic load [31]. It consists of 17 items asking for lifetime exposure to different psychologically traumatic events that were either personally experienced, witnessed, job-related, or individuals had heard about, thus following the DSM-5 definition of a psychologically traumatic event (items: (1) Natural disaster, (2) fire or explosion, (3) transportation accident, (4) serious accident at work, home, or during recreational activity, (5) exposure to toxic substances, (6) physical assault, (7) assault with a weapon, (8) sexual assault, (9) other unwanted or uncomfortable sexual experience, (10) combat or exposure to a war-zone, (11) captivity, (12) life-threatening illness or injury, (13) severe human suffering, (14) sudden violent death, (15) sudden accidental death, (16) serious injury, harm, or death caused to someone else by participant, (17) other stressful events). Participants were told that incidents that occurred during rescue work should be considered as job-related. Items were coded with yes/no and endorsed items were summed into two categories for personally experienced/witnessed events and for job-related events.

#### 2.3.2. PTSD

PTSD symptoms in the last seven days were measured according to the criteria in the eleventh revision of the International Classification of Diseases (ICD-11) [32], using the German version of the Impact of Event Scale-revised (IES-R) [33]. Differing from the English original, the German version uses a four-point Likert scale from 1 (“not at all”) to 4 (“often”). The IES-R is an often-applied screening tool and can be used to screen for ICD-11 PTSD by selecting six relevant items that match ICD-11 clusters of re-experiencing (unwanted pictures; upsetting dreams), avoidance (avoidance of reminders; avoidance of thoughts), and sense of current threat (being easily startled; being watchful) [34]. In the current study, this approach was applied and an overall score of PTSD symptom severity, as well as cluster-specific sum scores for re-experiencing, avoidance, and perception of current threat were calculated, respectively. Cronbach’s α was high with 0.8.

#### 2.3.3. Social Acknowledgment

Social support was measured using the German version of the Social Acknowledgment Questionnaire (SAQ) [35]. The SAQ assesses perceived social acknowledgment as a survivor. The questionnaire contains 16 items on a four-point Likert scale ranging from 0 (“I don’t agree at all”) to 3 (“I fully agree”). It consists of three distinct subscales that reflect aspects of social acknowledgment: Recognition (five items, e.g., “Many people offered their help in the first few days after the incident”), general disapproval (six items, e.g., “Somehow I am no longer a normal member of society since the incident”), and familial disapproval (five items, e.g., “My family finds my reaction to the incident to be exaggerated”). For recognition, lower values indicate less societal acknowledgment, whereas general and familial disapproval are interpreted in the opposite direction. Internal consistency in the study was moderate (Cronbach’s α = 0.73–0.76).

#### 2.3.4. Sense of Coherence

Sense of coherence was measured with the German version of the revised Sense of Coherence Scale (SOC-R) [36]. The scale contains 13 items and answers are rated on a five-point Likert scale from 1 (“not at all”) to 5 (“extremely”). It consists of three subscales that reflect dimensions of coherence: Manageability (ability to handle difficult situations, five items, e.g., “One can always find a way to cope with painful things in life”), balance (ability to balance positive and negative experiences and feelings, four items, e.g., “I am convinced that a lot of negative feelings (e.g., rage) also have positive sides”), and reflection (ability to understand connections or take on different perspectives, four items, e.g., “I always try to see things in context”). Internal consistency in the study was satisfactory (α = 0.71–0.81).

#### 2.3.5. Resilience

Resilience was measured using the German short version of the Resilience Scale, which assesses psychological resistance to adversity in the form of a personality trait (RS-11) [37]. The RS-11 consists of 11 items rated on a seven-point Likert scale ranging from 1 (“I don’t agree”) to 7 (“I completely agree”), e.g., “I usually manage one way or another”. Items were summed up to create an overall score. Internal consistency in the current study was high (Cronbach’s α = 0.93).

#### 2.3.6. Statistical Analysis

Data analyses followed a multi-step approach in order to reflect inter-relations between constructs. In a first step, ordinary least square multiple regression models were run with the three PTSD symptom clusters and overall symptom severity as outcomes. All variables that had shown significant or marginally significant bivariate correlations with the outcome (see Table S1) were considered as predictors. Next, two different graphical network models were applied in order to gain insights into complex inter-relations between variables. In network models, nodes represent variables, whereas edges reflect relations between variables. Weights of edges reflect the strengths of associations. First, Pairwise Markov random field (RMRF) Gaussian graph models were calculated. RMRF estimate and visualize reciprocal conditional dependencies between variables. The approach followed state-of-the-art recommendations [38]. Robustness was assessed by applying different bootstrapping methods (see online supportive material). Second, the results of RMRF models were compared to a Bayesian network model derived from directed acyclic graphs (DAG). In contrast to Gaussian graph models, DAGs can be used to investigate underlying pathways. The approach of combining RMRF with DAG models has been applied in a recent investigation in the field of Psychotraumatology [39] and followed state-of-the art recommendations [40]. Details are reported in Appendix A. Analyses were carried out with a complete case dataset using R [41] with the packages bootnet [38] and bnlearn [42], as well as their dependencies.

## 3. Results

Exposure to self-experienced and witnessed psychologically traumatic events was high (90%), followed by exposure to psychologically traumatic events within a job-related context (53%). Rates were highest for transportation accidents and other events (66% for both), severe human suffering (46%), other serious accidents (36%) or critical illnesses (36%) and natural disasters (35%). The minority reported a clinically relevant diagnosis of PTSD, according to ICD-11 (10%). All details have been extensively described elsewhere [30].

### 3.1. Regression Models

In the bivariate associations, the significant variables associated with almost all of the PTSD clusters were SAQ general disapproval, SAQ familial disapproval, and resilience. In addition, SOC-R manageability and SAQ recognition were negatively associated with symptoms of re-experiencing and perception of current threat, respectively (see Table S1). Significant variables were considered as predictors in the regression model. Controlling for both job-related, and overall, psychologically traumatic events, all regression models resulted in a similar pattern: Only SAQ general disapproval was positively related to overall PTSD symptom severity, and specifically to all clusters. Table 1 reports regression coefficients in detail.

### 3.2. RMRF Gaussian Graph and DAG Models

**Figure 1:**
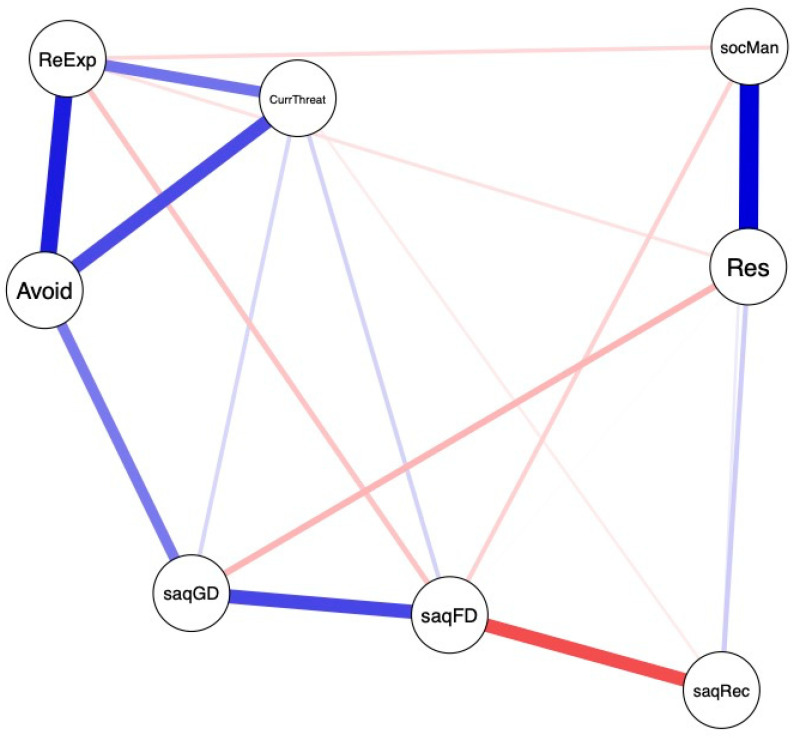
Estimated network of the RMRF Gaussian graph model. Note. ReExp = PTSD symptoms of re-experiencing, Avoid = PTSD symptoms of avoidance, CurrThreat = PTSD symptoms of perception of current threat, socMan = sense of coherence-revised subscale manageability, Res = resilience, saqGD = social acknowledgment subscale general disapproval, saqFD = social acknowledgment subscale familial disapproval, saqRec = social acknowledgment subscale recognition.

The RMRF Gaussian graphical model is depicted in Figure 1. Particularly strong associations were apparent among all three PTSD symptom clusters, among resilience, and SOC-R manageability, as well as among the SAQ familial disapproval and both SAQ recognition and SAQ general disapproval (all associations were observed in the intended positive or negative directions). These associations were also reflected in the centrality indices (strength, closeness, and betweenness) of the protective factors (see Figure S1). Based on all three indices, the most important variable in the model was SAQ general disapproval, followed by SAQ recognition.

Regarding edges between a potential protective factor and PTSD symptom clusters, only SAQ general disapproval was strongly associated with the PTSD cluster of avoidance. Much smaller edges were apparent among resilience and SAQ general disapproval, as well as among SAQ familial disapproval and PTSD symptoms of re-experiencing. However, the evaluation of model robustness indicated that the importance of the edges must be interpreted with care. Overlapping bootstrapped 95% CIs around edge weights implied instability in the order of edges (see Figure S2). Investigating the stability of the centrality indices indicated that strength and closeness remained more stable across subsets of cases, in contrast to betweenness (see Figure S3). Associated CIs for all correlations, except for strength, reflected large variability and the CS coefficients confirmed associations between the original sample and the subset sample values below the recommended threshold (all CS < 0.25). Bootstrapped difference tests between nodes resulted in no significant differences for any index.

**Figure 2:**
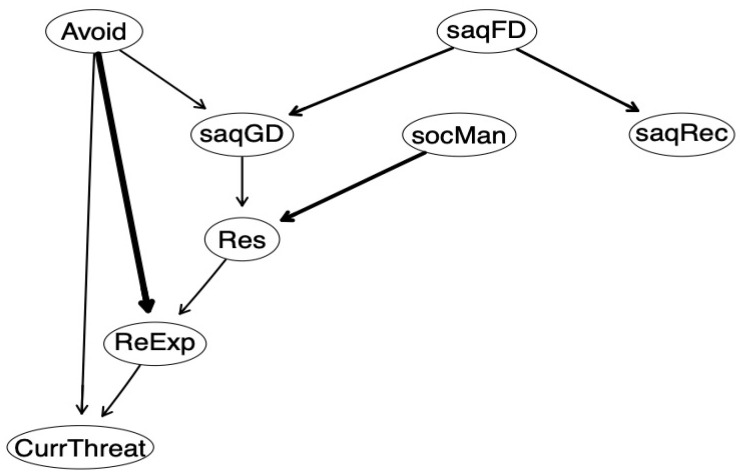
Estimated network of the Bayesian DAG model. Note. Edge (relation between variables) thickness reflects the importance of edges in the model regarding model fit. ReExp = PTSD symptoms of re-experiencing, Avoid = PTSD symptoms of avoidance, CurrThreat = PTSD symptoms of perception of current threat, socMan = sense of coherence-revised subscale manageability, Res = resilience, saqGD = social acknowledgment subscale general disapproval, saqFD = social acknowledgment subscale familial disapproval, saqRec = social acknowledgment subscale recognition.

With respect to the DAG Bayesian model, the averaged stable model indicated a more complicated picture of symptom exacerbation (see Figure 2). Similar to the Gaussian graph model, SOC-R manageability indirectly contributed to symptoms of re-experiencing via resilience, pointing to the importance of SOC-R manageability as a starting point for trauma-related distress via the pathway of resilience. In the DAG model, these paths were much more relevant than in the Gaussian graphical model. SAQ familial disapproval resulted in higher levels of SAQ general disapproval. This path, in turn, affected symptoms of PTSD re-experiencing via resilience. As in the RMRF, a path existed between SAQ general disapproval and PTSD avoidance, however, the direction indicated that SAQ general disapproval was a consequence of PTSD avoidance behavior. There was no other direct path from protective factors to any of the other PTSD symptom clusters. In contrast to assumptions about PTSD symptom development, avoidance led to increased symptoms in re-experiencing and current threat.

Checking probabilities for direction of path accuracy, focusing on protective variables, demonstrated the highest probability from resilience to PTSD re-experiencing, whereas almost all other path direction probabilities were in a similar range (see Figure S4).

## 4. Discussion

The current study presents the first empirical investigation of factors that may buffer the development of PTSD in canine search and rescue teams from Europe. In the regression analysis, the most important finding was that general disapproval presented the only association with PTSD and its clusters when controlling for trauma exposure. The finding is in line with earlier work identifying social support as a strong protective factor against PTSD in other heterogeneous rescue worker subgroups, e.g., [8,18,19], including search and rescue teams [24]. The study underlines the importance of social support for PTSD prevention in voluntary search and rescue dog handlers. When considering multivariate dependencies between several protective factors in the graph models, a more complex pattern emerged. The models indicated that familial disapproval was linked to both general disapproval and recognition and affected re-experiencing symptoms through general disapproval and resilience. The finding fits well with research showing that favorable social environments foster resilience [43]. The importance of familial support may also link to specific characteristics of the canine search and rescue sample. Since all engagement is voluntarily and carried out during personal time, support of close family members might play a particularly important role. This is in line with findings that a significant proportion of rescue workers experience stigma [8], which may also arise from their own families, thus compromising social support. The role of social support may be particularly important for voluntary dog handlers, as previous research suggests that dog affinity can enhance perceived social support in pet owners [20,21].

In addition, we found that the manageability facet of sense of coherence was related to lower PTSD symptoms. The result conforms to previous research on other rescue worker professions [12,16]. In the current study, manageability indirectly affected symptoms of re-experiencing via resilience. The finding confirms previous evidence of an association between sense of coherence and resilience [15] and corresponds to the assumption that sense of coherence presents an overlapping aspect of resilience [44]. It is also consistent with research on other rescue groups that observed associations between resilience and PTSD [7,12,13]. The conviction of being able to cope with difficult situations might result in higher levels of resilience among dog handlers, which, in turn, may prevent the development of re-experiencing symptoms. The finding is supported by observations by Alvarez and Hunt [20] that efficient training results in lower symptom severity in canine search and rescue teams, suggesting that the feeling of being prepared adds to resilience. The results of our study could argue in favor of resilience being a dynamic personal resource that is influenced by the belief of being able to manage and cope with difficult situations, as well as by facets of social acknowledgment. Further research is needed to shed light on the specific role of resilience in the relationship between manageability, general disapproval, and levels of re-experiencing.

Inconsistent with assumptions about PTSD symptom development and with earlier results on the effect of social support on PTSD symptom development, the DAG model indicates that avoidance influences perception of current threat and re-experiencing symptoms, as well as general disapproval (c.f. [3]). DAG models do not reflect cycles or feedback loops of symptom exacerbation, but rather illustrate directions that contribute most to the model fit. Consequently, relationships between PTSD symptom clusters may include feedback loops, and similar observations have been reported in other PTSD network studies [39].

When comparing the two network models, a broadly similar pattern emerged regarding nodes and associations. Manageability and resilience were only related to symptoms of re-experiencing in the DAG model. Since this method is not yet well established, future research is needed to investigate the stability of DAG models.

The strength of the study lies in the multi-step approach of increasing statistical complexity that allows conclusions to be drawn about complex inter-relations between variables. Furthermore, the DAG model offers the opportunity to investigate potential pathways. The findings underline the need for approaches targeting social support and individual cognitive factors in preventing PTSD in rescue dog handlers. Strengthening resilience by focusing on functional coping styles in response to adverse events seems promising and thorough preparation in terms of greater manageability of events might enable individuals to overcome deployment-related traumatic stress. The findings also demonstrate the importance of incorporating multivariate associations between variables beyond more conventional analyses in order to best reflect patterns and derive comprehensive conclusions.

### Limitations

The current study includes several limitations. The sample size was small, and an online convenience sample was used. This may have compromised generalizability and resulted in insufficient power (see [30] for a detailed discussion). The use of self-report measures and screening tools may overestimate symptom presence. Furthermore, the cross-sectional design does not allow for the derivation of causal conclusions regarding pathways. DAG models allow valuable first insights into potential patterns of directions. However, definite conclusions on the direction cannot be drawn. In light of model instability, strength of associations and pathways warrant further research, particularly using longitudinal research designs. Model stability indices (CS-coefficients) were below the recommended cut-off value of 0.25, and thus, only small and potentially unstable effects can be assumed. It is possible that this threshold is too conservative, since previously published network models for PTSD have reported comparable values, e.g., [39,45,46]. The network analytical approach, in general, has been criticized due to low replicability across samples, and there is an ongoing debate about its clinical utility, e.g., [46]. This study provides the first insight into potential mechanisms for buffering PTSD among voluntary dog handlers. Future research is warranted to replicate findings and confirm model stability. While the current study did not assess the comparison of rescue workers accompanied by dogs with those from the same subgroup deployed without canine assistance, this could provide additional valuable insight into the impact of dog presence during deployment. Finally, the current study did not consider the relationship quality between handlers and dogs. Relationship quality has been shown to have an important impact on perceived social support, such that individuals with either very weak or very strong human–dog bonds may show lower resilience [21]. This relationship quality may also influence other coping factors, such as manageability. Future research should further elaborate on human–dog interactions and the relevance for preventing PTSD in canine search and rescue teams.

## 5. Conclusions

The present study stresses the importance of social support for mental health in canine search and rescue teams and presents the first insights into complex interactions between cognitive coping styles. The findings suggest the need for a multifaceted preventive approach focusing on aspects that are often neglected, such as resilience, manageability of events, and social support. The current study serves as groundwork for further research into this under-represented sample.

## Figures and Tables

**Table 1:** Linear regression models investigating associations between predictor variables and PTSD symptom severity and symptom clusters.

Variable	*β (SE)*	*p*
**PTSD Symptom Severity**		
Resilience	−0.05 (0.04)	0.24
SAQ General Disapproval	**0.62 (0.19)**	<0.01 **
SAQ Familial Disapproval	−0.06 (0.17)	0.74
*F*(5/79) = 6.5, *p* < 0.001 ***, *Adjusted R*^2^ = 0.25		
**PTSD Cluster Re-Experiencing**		
Resilience	−0.02 (0.02)	0.33
SAQ General Disapproval	**0.14 (0.07)**	<0.05 *
SOC-R Manageability	−0.06 (0.07)	0.37
*F* (5/82) = 4.2, *p* < 0.01 **, *Adjusted R*^2^ = 0.15		
**PTSD Cluster Avoidance**		
Resilience	−0.05 (0.04)	0.24
SAQ General Disapproval	**0.62 (0.19)**	<0.01 **
SAQ Familial Disapproval	−0.06 (0.17)	0.74
*F*(5/79) = 6.5, *p* < 0.001 ***, *Adjusted R*^2^ = 0.25		
**PTSD Cluster Perception of Current Threat**		
SAQ General Disapproval	**0.14 (0.06)**	<0.05 *
SAQ Familial Disapproval	0.03 (0.06)	0.62
SAQ Recognition	−0.05 (0.04)	0.28
*F* (5/72) = 3.7, *p* < 0.01 **, *Adjusted R^2^* = 0.15		

Note. Intercepts were removed due to enhanced readability. All analyses controlled for self-experienced/witnessed and job-related exposure to psychologically traumatic events. β = standardized regression coefficient; SE = standard error; * *p* < 0.05; ** *p* < 0.01; *** *p* < 0.001. Significant values are printed in bold.

## Supplementary Materials

The following are available online at https://www.mdpi.com/1660-4601/17/17/6184/s1, Figure S1: Centrality indices for the Gaussian graph model, Figure S2: Bootstrapped weights of edges (95% CI). The original sample values are printed in red, bootstrapped values are printed in black. Gray areas indicate 95% confidence intervals, Figure S3: Average correlation between original centrality indices and their values resulting from random case-dropping bootstrapping. Percentage of dropped cases is indicated on the x-axis, Figure S4: Averaged DAG model with path thickness indicating direction probabilities. Table S1: Bivariate associations between PTSD symptom clusters, overall symptom severity and protective factors (SOC-R, SAQ, RS-11) were investigated by calculating Pearson’s Product-Moment correlations.

## Appendix A. Details Regarding the Statistical Analyses

**Step 1.** Regression models were controlled for exposure to both job-related and overall psychologically traumatic events. Since levels of PTSD did not differ between men and women (*t*(66.23) = −0.8, *p* = 0.42) and also age was not associated with PTSD symptom severity (r = 0.10, *p* = 0.32), analyses were not further controlled for socio-demographic variables.

**Step 2a.** For the Gaussian graph models, the least absolute shrinkage and selection operator (LASSO) was used. It penalizes numbers of associations between variables and favors sparse models. Thus, it is suitable for small sample sizes. The degree of penalization was controlled by minimizing the Extended Bayesian Information Criterion (EBIC). Furthermore, a non-paranormal transformation was applied to control for skewed data before estimating the network. Node importance was investigated with z-standardized centrality indices: Node strength (direct connection between nodes by estimating their weights), closeness (indirect connection between nodes), and betweenness (indirect importance of one node in the path between two other nodes). In order to investigate model accuracy, general recommendations were followed: First, robustness of weight of edges was investigated with 95% Confidence Intervals (CI) derived from non-parametric bootstrapping with 2500 resamples. Second, case-dropping bootstrapping was used to calculate stability of centrality indices [32,33]. Third, a correlation coefficient (CS) between the original indices and indices derived from bootstrapping was calculated. CS ≥ 0.25 is required to assume robustness, see [32,33]. Finally, bootstrapped difference tests were applied to investigate significant differences in nodes and edges.

**Step 2b.** For the Bayesian DAG model, the hill-climbing algorithm was applied. In order to avoid local minima, procedures were randomly restarted (10×) with 50 perturbations. In addition, bootstrapping of 2500 samples was performed. The final model consisted of an average of all bootstrapped models. Minimizing the Bayesian Information Criterion (BIC) was used as criterion to assess strength of edges. Edges for the final model were selected according to a statistically indices as outlined in [39]. Further details and overall recommendations are reported in [33,34].
