# Supplementary material for: Buffering PTSD in Canine Search and Rescue Teams? Associations with Resilience, Sense of Coherence, and Societal Acknowledgment
Source: Int J Environ Res Public Health. 2020 Aug 26;17(17):6184. doi: 10.3390/ijerph17176184 (PMC7503916; doi:10.3390/ijerph17176184)
Supplement: Supplementary file 1 [file ijerph-17-06184-s001.pdf]

Table S1. Bivariate associations between PTSD symptom clusters, overall symptom severity and protective factors.

PTSD symptoms	SOC-R Manage	SOC-R Balance	SOC-R Reflection	Res	SAQ Recognition	SAQ Gen Disapproval	SAQ Fam Disapproval
**PTSD Overall**	−0.16	−0.04	−0.10	**−0.27 ****	−0.15	**0.42 *****	**0.26 ***
**PTSD Re-Experiencing**	**−0.21 ***	−0.06	−0.17	**−0.27 ****	−0.06	**−0.31 ****	0.07
**PTSD Avoidance**	−0.16	−0.07	−0.04	**−0.26 ***	−0.15	**0.44 *****	**0.33 ****
**PTSD Threat**	−0.02	0.05	−0.05	−0.14	**−0.21 ***	**0.34 ****	**0.30 ****

Note. SOC-R = Sense of coherence revised, Res = Resilience, SAQ = Social Acknowledgement. Gen Disapproval = General Disapproval, Fam Disapproval = Familial Disapproval, PTSD = Posttraumatic Stress Disorder, * $p < 0.05$, ** $p < 0.01$, *** $p < 0.001$. Significant values are printed in bold.

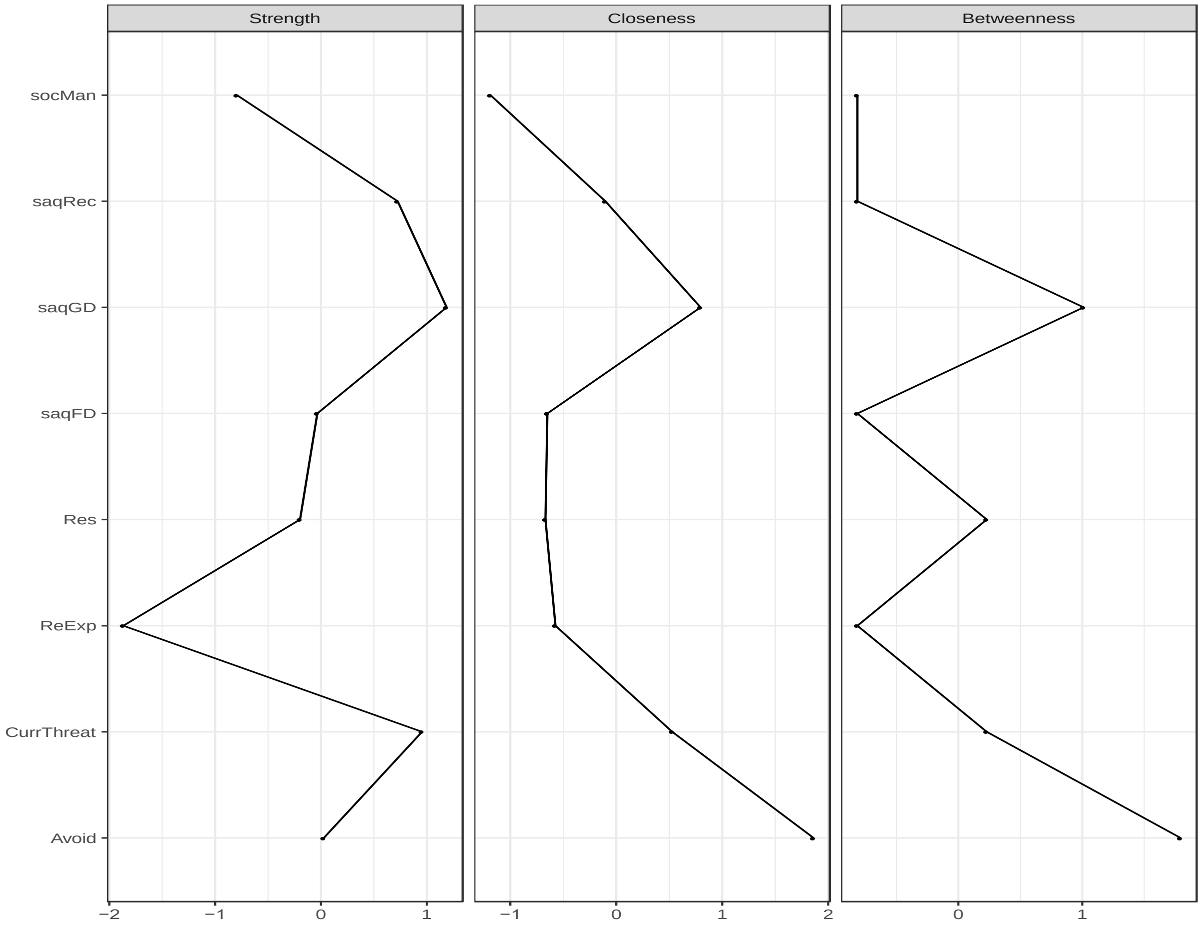

Figure S1. Centrality indices for the Gaussian graph model.

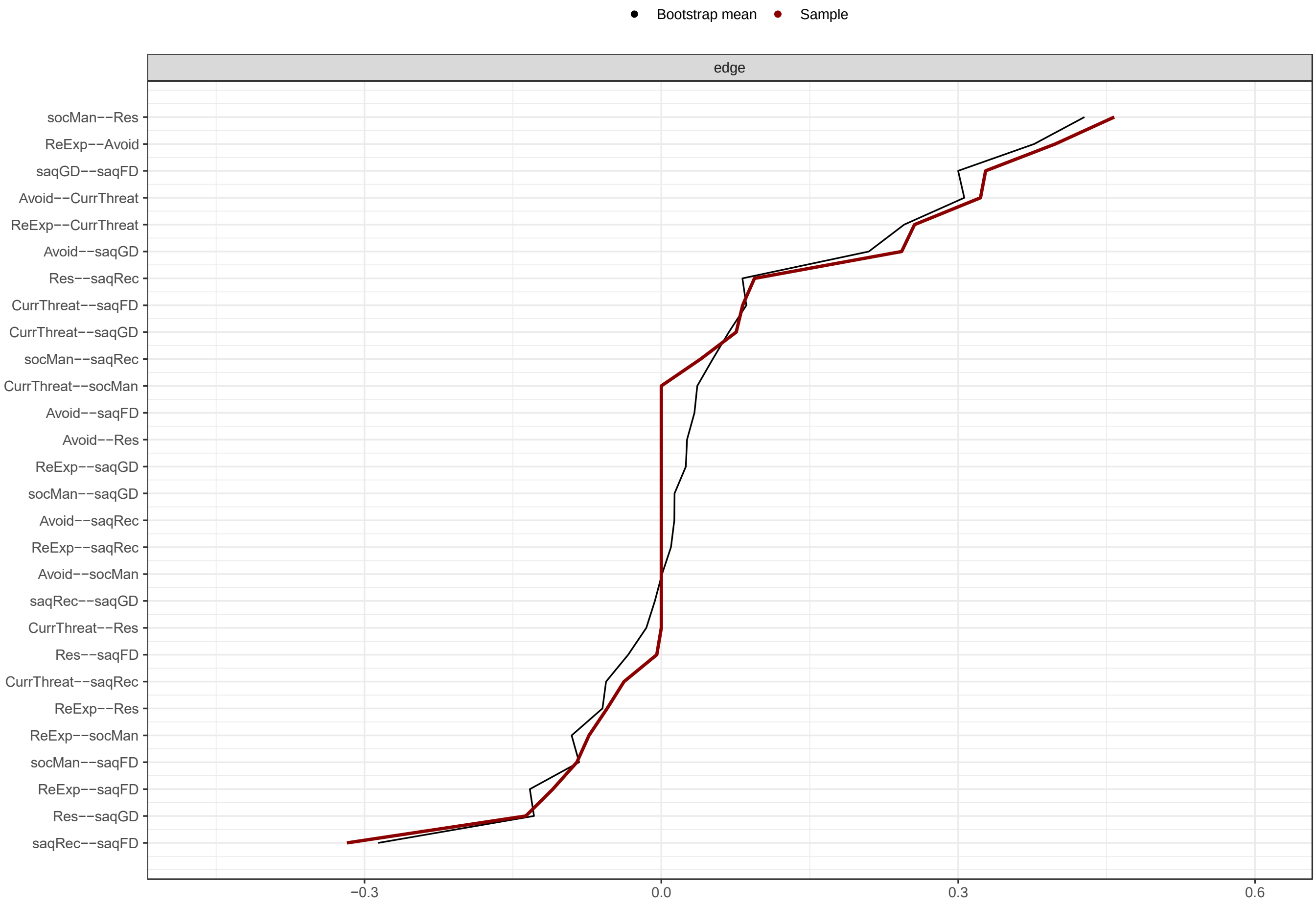

Figure S2.: Bootstrapped weights of edges (95% CI)

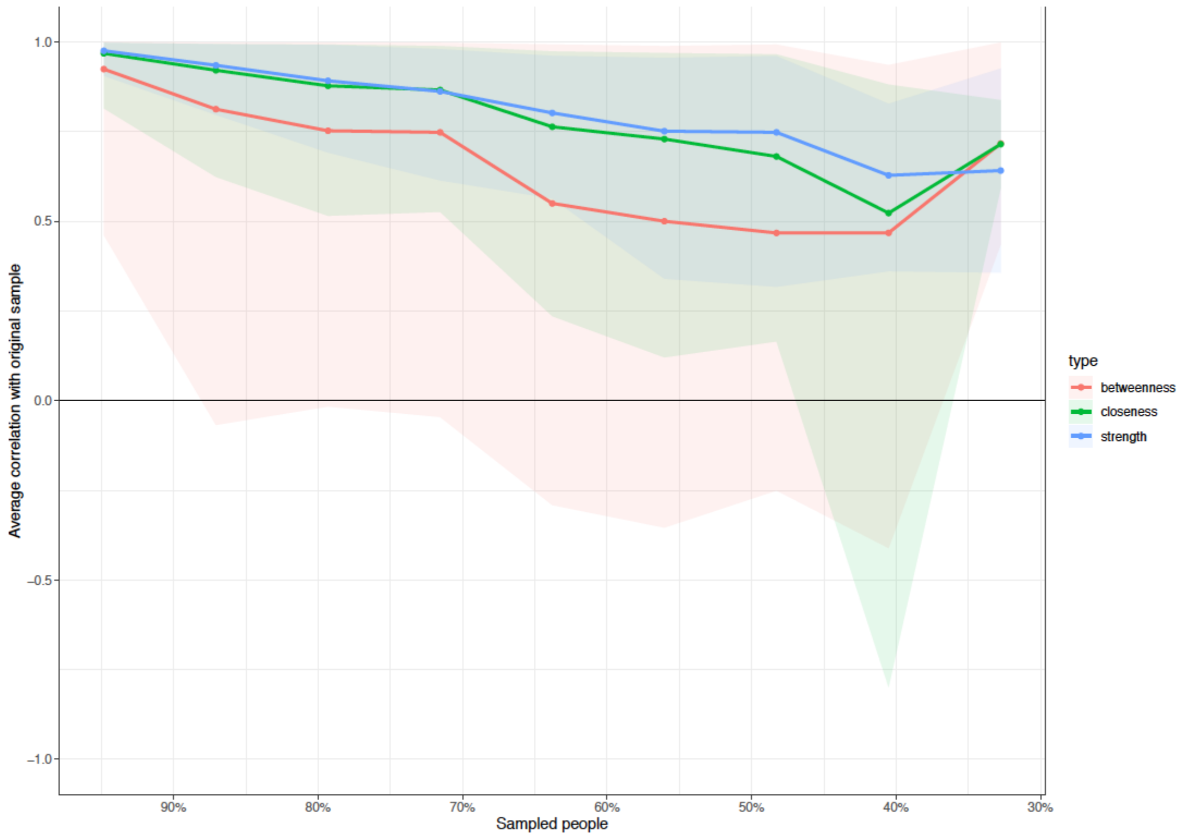

Figure S3. Average correlation between original centrality indices and their values resulting from random case-dropping bootstrapping.

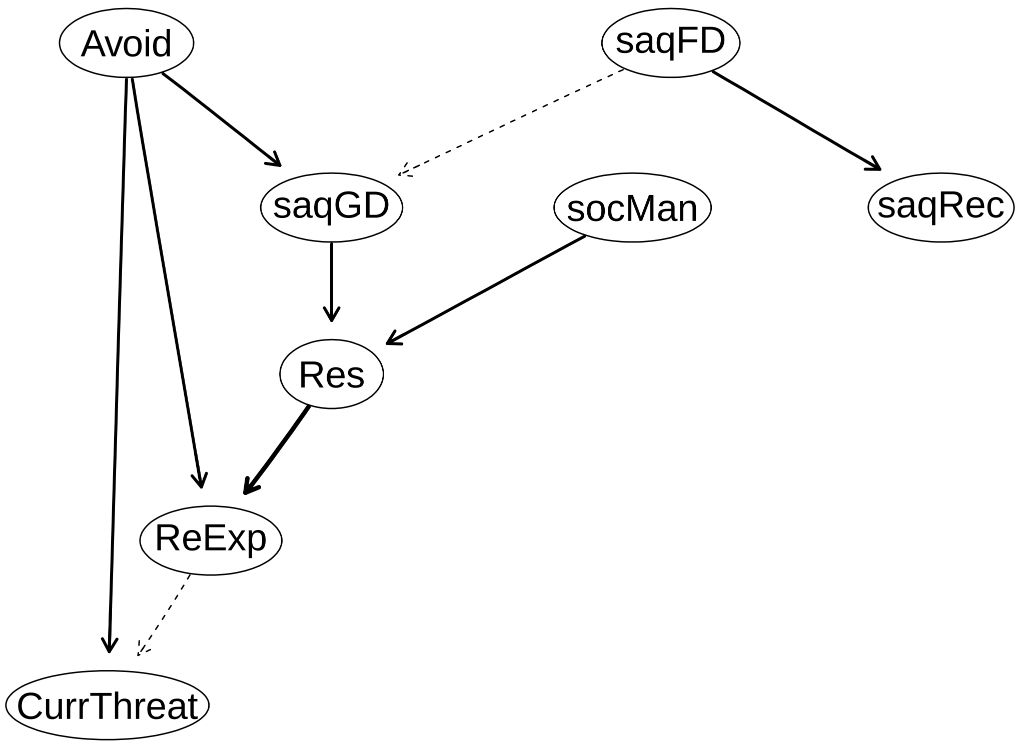

Figure S4. Averaged DAG model with path thickness indicating direction probabilities. ReExp = PTSD symptoms of re-experiencing, Avoid = PTSD symptoms of avoidance, CurrThreat = PTSD symptoms of perception of current threat, socMan = sense of coherence-revised subscale manageability, Res = resilience, saqGD = social acknowledgment subscale general disapproval, saqFD = social acknowledgment subscale familial disapproval, saqRec = social acknowledgment subscale recognition, PTSD = Posttraumatic Stress Disorder.
